# Dissociating different temporal stages of emotional word processing by feature-based attention

**DOI:** 10.1038/s41598-023-43794-4

**Published:** 2023-10-06

**Authors:** Sebastian Schindler, Ria Vormbrock, Hanne Helming, Thomas Straube

**Affiliations:** 1 Institute of Medical Psychology and Systems Neuroscience, University of Muenster, Von-Esmarch-Str. 52, 48149 Münster, Germany; 2 Otto Creutzfeldt Center for Cognitive and Behavioral Neuroscience, University of Muenster, Münster, Germany

**Keywords:** Neuroscience, Cognitive neuroscience, Emotion, Motivation, Sensory processing, Psychology

## Abstract

Negative emotional content is prioritized across different stages of information processing as reflected by different components of the event-related potential (ERP). In this preregistered study (*N* = 40), we investigated how varying the attentional focus allows us to dissociate the involvement of specific ERP components in the processing of negative and neutral words. Participants had to discriminate the orientation of lines overlaid onto the words, the word type (adjective/noun), or the emotional content (negative/neutral). Thus, attention was either not focused on words (distraction task), non-emotional aspects, or the emotional relevance of words. Regardless of the task, there were no significant differences between negative and neutral words for the P1, N1, or P2 components. In contrast, interactions between emotion and task were observed for the early posterior negativity (EPN) and late positive potential (LPP). EPN differences were absent during the distraction task but were present in the other two tasks. LPP emotion differences were found only when attention was directed to the emotional content of words. Our study adds to the evidence that early ERP components do not reliably separate negative and neutral words. However, results show that mid-latency and late stages of emotion processing are separable by different attention tasks. The EPN represents a stage of attentional enhancement of negative words given sufficient attentional resources. Differential activations during the LPP stage are associated with more elaborative processing of the emotional meaning of words.

## Introduction

Language is abstract and arbitrary, while even single words differ in their emotional quality. Event-related potential (ERP) studies show that our brains differentiate between negative and neutral words, even when emotion is not directly relevant to the experiment^1–6^. However, it has been recently shown that emotional information processing crucially depends on the focus of attention, enabling a dissociation of ERP unfolding across the time course of stimulus processing.

Different components of the ERP are associated with different stages of information processing. They can broadly be distinguished in rather early components (such as the P1 and N1), mid-latency (such as the P2, Early Posterior Negativity, EPN), and late components (such as the Late Positive Potential, LPP). Among early ERPs, the P1 (~ 80 to 100 ms post-stimulus) is an occipitally scored positivity, followed by the N1 (~ 120 to 170 ms) as occipito-temporal negativity. Both components reflect the early stages of stimulus processing^7,8^. They are related to early stimulus gain processes^9,10^, and are strongly influenced by low-level visual information^11–13^. Concerning mid-latency ERPs, the P2 component is a positive polarization and peaks at around 200 ms, with a more variable distribution, sometimes distinguishing an anterior and posterior P2 component^14^. The anterior P2 has been more closely related to exogenous attention^14,15^. Word repetition has been shown to increase the P2^16^. The early posterior negativity (EPN) is observed as differential occipital-temporal negativity when contrasting emotional and neutral stimuli and is typically observed between 200 and 300 ms. The EPN is related to early emotional tagging and attention processes toward relevant information^2,17,18^. Among late ERPs, the late positive potential (LPP) is part of the family of late positivities, emerging from approximately 400 ms onwards up to seconds after stimulus appearance. It is identified by contrasting emotional and neutral stimuli and indicates stimulus evaluation and controlled attention processes, with a centro-parietal topographic distribution, but this varies and seems to depend on stimulus types, and tasks^19,20^.

Concerning early emotion effects and their modulation by attention asks, effects are mixed for the P1. Some studies found larger amplitudes for negative compared to neutral words^21^, with some only in males^22^ samples or the left hemisphere^23^, while other studies reported decreased P1 amplitudes for negative compared to neutral words, depending on target relevance or word frequency^3,24^, and other studies did not find differences^4,12,25–31^. No differential effects concerning negative valence were observed between an emotional vs. color judgment task^4^, when attending to the lexical or emotional information^26^, when performing a lexical decision task rather than reading^29^, or when instructed to inhibit a word^32^. For the N1, several studies reported larger N1 amplitudes for negative than neutral words^33,34^, sometimes restricted in the left hemisphere^17,23^. Nevertheless, other studies found emotion effects depending on word frequency^3^ or target status^24^. Some studies reported effects restricted to the right hemisphere, or only in response to positive words^26^, or the absence of effects^12,25,30,35^. The N1 to negative as compared to neutral words was not modulated by emotional vs. color judgment tasks^4,36,37^, or when attending to lexical vs. emotional information^26,29^. For the following P2, increased P2 amplitudes for negative words have been observed^31,37,38^, sometimes being left- or right-lateralized^15,36^, or descriptively larger for concrete negative words^39^. Other studies reported effects only for positive (concrete) words or no effects for negative words^1,40^. Thus, early emotion effects differ considerably between studies and may reflect differences in the used languages, specific stimulus sets, and attention tasks. Systematic studies with large sample sizes, well-controlled stimuli, and variation of task conditions are strongly needed.

More reliably, effects for emotional words are found during mid-latency and late processing stages. The EPN arises at about 200 ms and is related to early lexical access^17^, perceptual tagging^2^, and attention processes^41^. The LPP occurs from about 400 ms after presenting a word and reflects later stages of attention, stimulus evaluation, and episodic memory encoding^25,33,42^. Nevertheless, several studies showed for negative words either no EPN^43–45^ or no LPP effects^26,27,35^. Effects seem to depend on attentional conditions. For example, EPN effects were present for tasks requiring emotional judgments but not when attending to the color of stimuli^4^. Hinojosa et al.^44^ showed a similar EPN pattern, although not significant for negative words. Following these studies, attention to the semantic meaning seems necessary to elicit mid-latency emotion effects in words. Hinojosa and colleagues^44^ also showed LPP effects for negative words when participants had to identify a word among pseudowords (i.e., had to attend to the meaning) but not when words had to be identified among non-recognizable stimuli. In this regard, late emotion effects for negative words were absent in several studies during structural (font consistency)^46^, color^37^, lexical^3^, or semantic^26^ tasks. Further, for the LPP, a study reported increasing effects when negative words were target-relevant as compared to neutral words^47^, but see^48^. This pattern of findings would be in line with a recent meta-analysis across different visual stimuli (with smaller effect sizes for word stimuli) that reported no reliable late amplitude effects during non-emotional tasks (e.g., watching, reading, or classification according to non-emotional attributes), but reliable effects during explicit emotion decision tasks^49^. However, as pointed out above, there are several conflicting findings, mostly due to studies that report late emotion effects during color, lexical, or semantic tasks^1,4,40,46,50^. This might be explained by attentional spillover to task-irrelevant word features.

To reduce the variability in experimental conditions and to better differentiate processing stages during emotion processing, we recently developed a design that systematically varied feature-based attention to emotional visual stimuli^51^. Here, participants pay attention to a stimulus-unrelated feature (e.g., overlaid thin lines), to the stimulus (e.g., specific emotion irrelevant stimulus features), or to the emotional meaning (e.g., negative or neutral content). Studies using faces or complex scenes showed dissociable modulations of the EPN and LPP across attention tasks^51–53^. Emotional EPN effects were absent in the perceptual but present in the other tasks, while LPP differences were only present when attention was directed to the emotional information^51–53^. For pictures and faces, increased N1/N170 responses were found regardless of task^51–53^. Thus, this kind of task allows the separation of more automatic (early) processing stages from subsequent mid-latency and late stages, which require sufficient attentional resources or task relevance during the processing of emotional stimuli. Importantly, the dissociation between ERP components requires brief presentation times to avoid attentional spillover to task-irrelevant stimulus features^54^. It remains an open question whether a similar dissociation of processing stages can also be revealed while processing negative vs. neutral words.

Different stages of emotion processing are dissociated by tasks that systematically increase attention to emotionally relevant stimulus features. To test how modulations depend on the given attended feature in word stimuli, we used three different tasks during which negative and neutral words (adjectives and nouns) were presented and examined differential responses across the whole processing stream (P1, N1, P2, EPN, and LPP). Participants had to decide (1) if the overlaid line orientation was either horizontal or vertical, (2) if the word was a noun or an adjective, or (3) if the word valence was negative or neutral. We expected that the later the component of the ERP, the stronger the increasing attention to emotionally relevant information would increase emotion effects (for the detailed registration, see https://osf.io/nrmsb). We explored emotion effects and interactions with the task for earlier ERPs (P1, N1, and P2). Concerning registered effects, we expected the EPN to increase amplitudes for negative words in the grammatical (adjective/noun) and emotion decision tasks compared to the perceptual task. Finally, LPP emotion effects should be increased in the emotion decision task compared to grammatical and perceptual decision tasks.

## Results

### Behavior

Regarding hit rate, the number of correct choices was not affected by emotion (*F*_(1,39)_ = 1.37, *p* = 0.250, η_P_^2^ = 0.034), but by task (*F*_(2,78)_ = 55.55, *p* < 0.001, η_P_^2^ = 0.588), with higher accuracy for the perceptual compared to both the grammatical (*p* = 0.048) and the emotion task (*p* < 0.001). The grammatical task also elicited higher accuracy than the emotion task (*p* < 0.001). Emotion and task did not interact (*F*_(1.30,50.56)_ = 2.49, *p* = 0.113, η_P_^2^ = 0.060; see Table 1). Regarding reaction times, main effects of emotion (*F*_(1,39)_ = 15.83, *p* < 0.001, η_P_^2^ = 0.289) and of task were identified (*F*_(2,78)_ = 91.91, *p* < 0.001, η_P_^2^ = 0.702), but no significant emotion by task interaction (*F*_(1.32,51.40)_ = 2.43, *p* = 0.117, partial η^2^ = 0.059). Reaction times were significantly shorter for negative than neutral words (*p* < 0.001) and shorter for the perceptual compared to both the grammatical and compared to the emotion task (*ps* < 0.001). The grammatical task also elicited shorter reaction times than the emotion task (*p* < 0.001).

**Table 1:** Behavioral results across the three attention tasks.

	Perceptual task		Grammatical task		Emotion task	
	Negative words	Neutral words	Negative words	Neutral words	Negative words	Neutral words
Accuracy (*SD*)	0.93	0.92	0.91	0.90	0.83	0.86
	(0.07)	(0.06)	(0.06)	(0.06)	(0.10)	(0.08)
Reaction time in ms (*SD*)	589	591	718	730	772	798
	(73)	(74)	(100)	(100)	(105)	(103)

Hits are displayed in proportion correct. Reaction times are rounded to milliseconds.

### Event-related potentials

For mean amplitudes of all examined ERPs, see Table 2 below. For hemisphere effects for the P1, N1, and EPN, see respective control analyses below in "Analyses of hemispheric differences in early emotion effects" section.

**Table 2:** Mean amplitudes for all ERPs across the three attention tasks.

	Perceptual task		Grammatical task		Emotion task	
	Negative words	Neutral words	Negative words	Neutral words	Negative words	Neutral words
P1 (*SD*)	3.11	2.95	2.86	2.87	2.87	2.93
	(1.99)	(1.79)	(1.80)	(1.94)	(1.65)	(1.96)
N1 (*SD*)	− 2.36	− 2.59	− 3.01	− 2.91	− 3.02	− 3.09
	(1.57)	(1.75)	(1.95)	(2.01)	(1.75)	(1.65)
P2 (*SD*)	1.01	1.21	1.43	1.38	1.52	1.41
	(1.19)	(1.23)	(1.23)	(1.32)	(1.17)	(1.18)
EPN (*SD*)	− 2.17	− 2.37	− 2.41	− 2.11	− 2.57	− 2.36
	(1.89)	(1.86)	(1.88)	(1.88)	(1.92)	(1.99)
LPP (*SD*)	2.07	2.07	2.08	1.86	2.08	1.41
	(1.68)	(1.73)	(1.85)	(1.75)	(1.75)	(1.76)

Mean amplitudes are displayed in microvolts, averaged across the respective time windows and sensors of interest (see methods section below).

#### P1 (90–110 ms)

**Figure 1:**
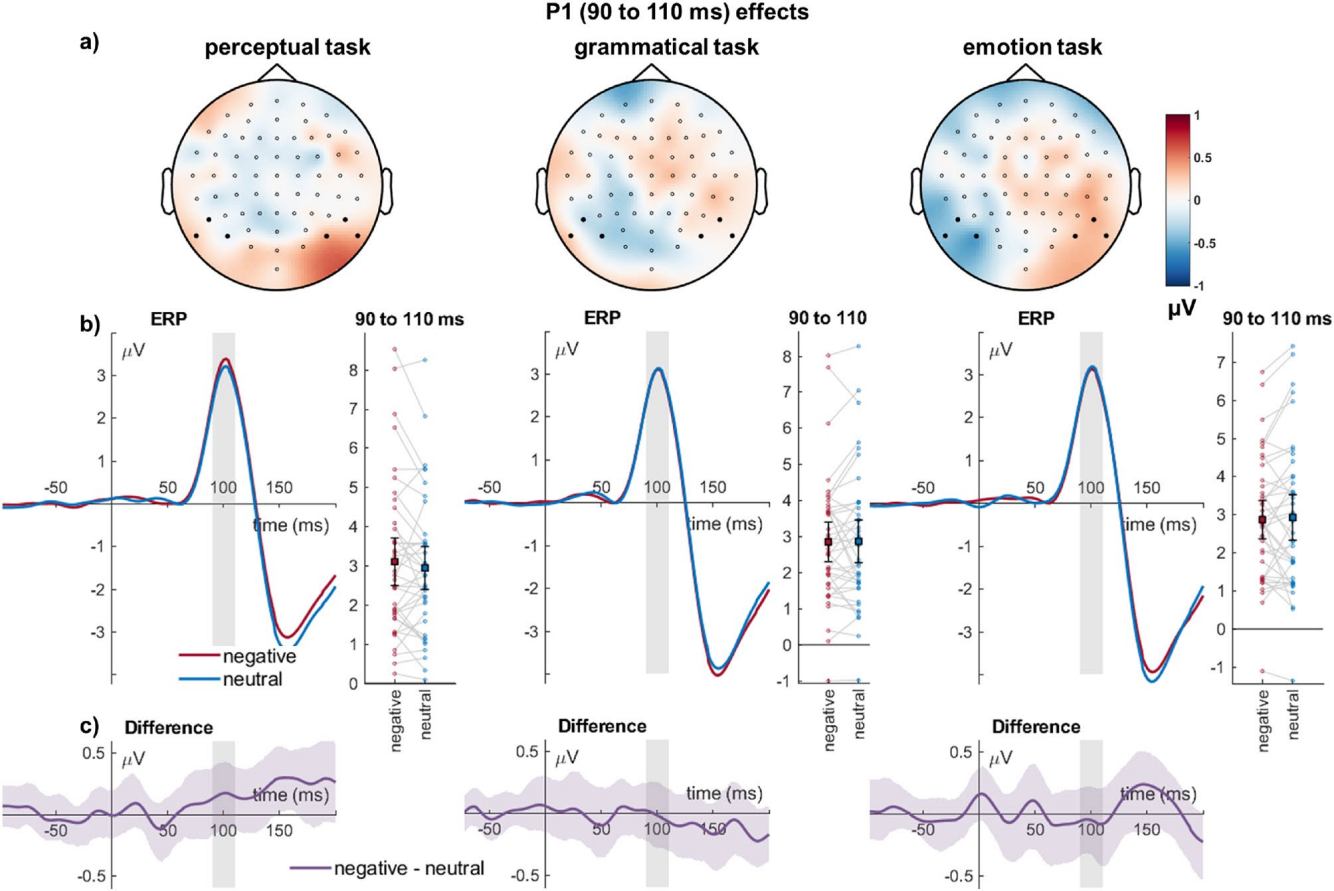
Occipital cluster showing P1 effects. (**a**) Scalp topographies depict the differences between negative and neutral words. (**b**) ERP waveforms show the time course over highlighted sensors. For bar plots, error bars show 95% confidence intervals for amplitudes averaged across selected sensors. Lines connect individual data points. (**c**) Respective difference plots displayed below contain 95% bootstrap confidence intervals of intra-individual differences.

For the P1, there were no main effects of emotion (*F*_(1,39)_ = 0.16, *p* = 0.696, η_P_^2^ = 0.004, see Fig. 1), and of task (*F*_(2,78)_ = 1.20, *p* = 0.306, η_P_^2^ = 0.030), and no interaction of emotion and task (*F*_(2,78)_ = 0.74, *p* = 0.483, η_P_^2^ = 0.019).

#### N1 (140–180 ms)

For the N1, there was no main effect of emotion (*F*_(1,39)_ = 1.09, *p* = 0.303, η_P_^2^ = 0.027). There was a main effect of task (*F*_(2,78)_ = 12.63, *p* < 0.001, η_P_^2^ = 0.245), with larger N1 amplitudes for both the emotion and grammatical task compared to the perceptual task (*ps* < 0.001), the former two conditions not differing from each other (*p* = 0.425). There was no interaction of emotion and task (*F*_(2,78)_ = 1.96, *p* = 0.148, η_P_^2^ = 0.048).

#### Early posterior negativity (200–350 ms)

**Figure 2:**
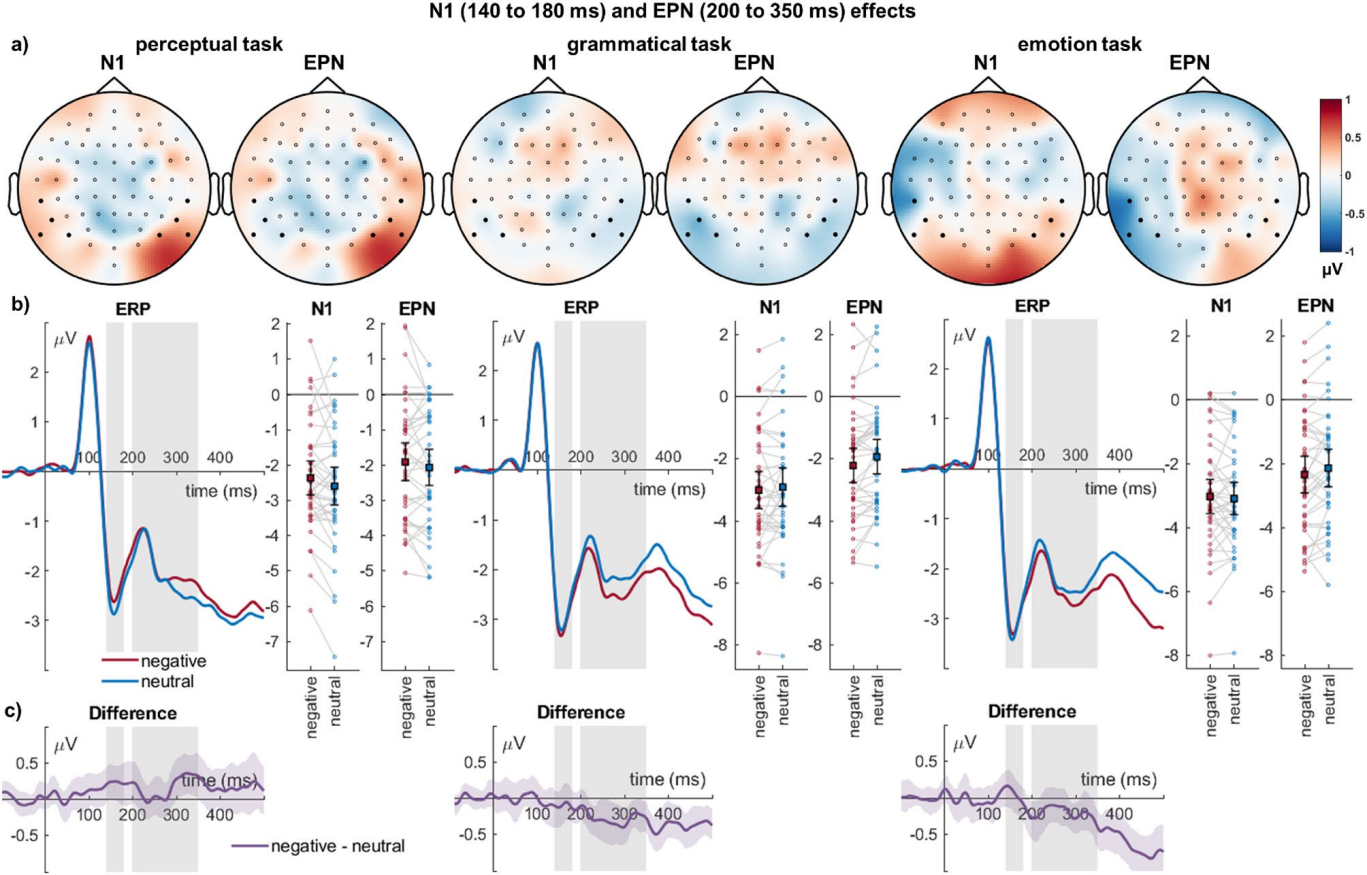
Occipito-temporal cluster showing N1 and EPN effects. (**a**) Scalp topographies depict the differences between negative and neutral words. (**b**) ERP waveforms show the time course over highlighted sensors. For bar plots, error bars show 95% confidence intervals for amplitudes averaged across selected sensors. Lines connect individual data points. (**c**) Respective difference plots displayed below contain 95% bootstrap confidence intervals of intra-individual differences.

For the EPN, there was no main effect of emotion (*F*_(1,39)_ = 3.24, *p* = 0.080, η_P_^2^ = 0.077), and no main effect of task (*F*_(2,78)_ = 1.39, *p* = 0.256, η_P_^2^ = 0.034). There was an interaction of emotion and task (*F*_(2,78)_ = 5.07, *p* = 0.008, η_P_^2^ = 0.115, see Fig. 2). Importantly, emotion differences were larger for the emotion compared to the perceptual task (*M*_difference_ =  − 0.36, *SD* = 0.90, *t*_(39)_ =  − 2.54, *p* = *0.0*15), and for the grammatical compared to the perceptual task (*M*_difference_ =  − 0.44, *SD* = 0.94, *t*_(39)_ =  − 2.96, *p* = *0.0*05). Negative-neutral differences did not differ between the emotion and grammatical tasks (*M*_difference_ = 0.08, *SD* = 0.96, *t*_(39)_ = 0.54, *p* = *0.5*95).

#### P2 (150–200 ms)

Similar to N1 effects, the central P2 was not affected by emotion (*F*_(1,39)_ = 0.06, *p* = 0.812, η_P_^2^ = 0.001), but by task (*F*_(2,78)_ = 7.53, *p* < 0.001, η_P_^2^ = 0.162). There were larger P2 amplitudes for both the emotion and grammatical task compared to the perceptual task (*ps* < 0.01), the former two conditions not differing from each other (*p* = 0.463). There was no interaction of emotion and task (*F*_(2,78)_ = 2.43, *p* = 0.095, η_P_^2^ = 0.059).

#### Late positive potential (380–800 ms)

**Figure 3:**
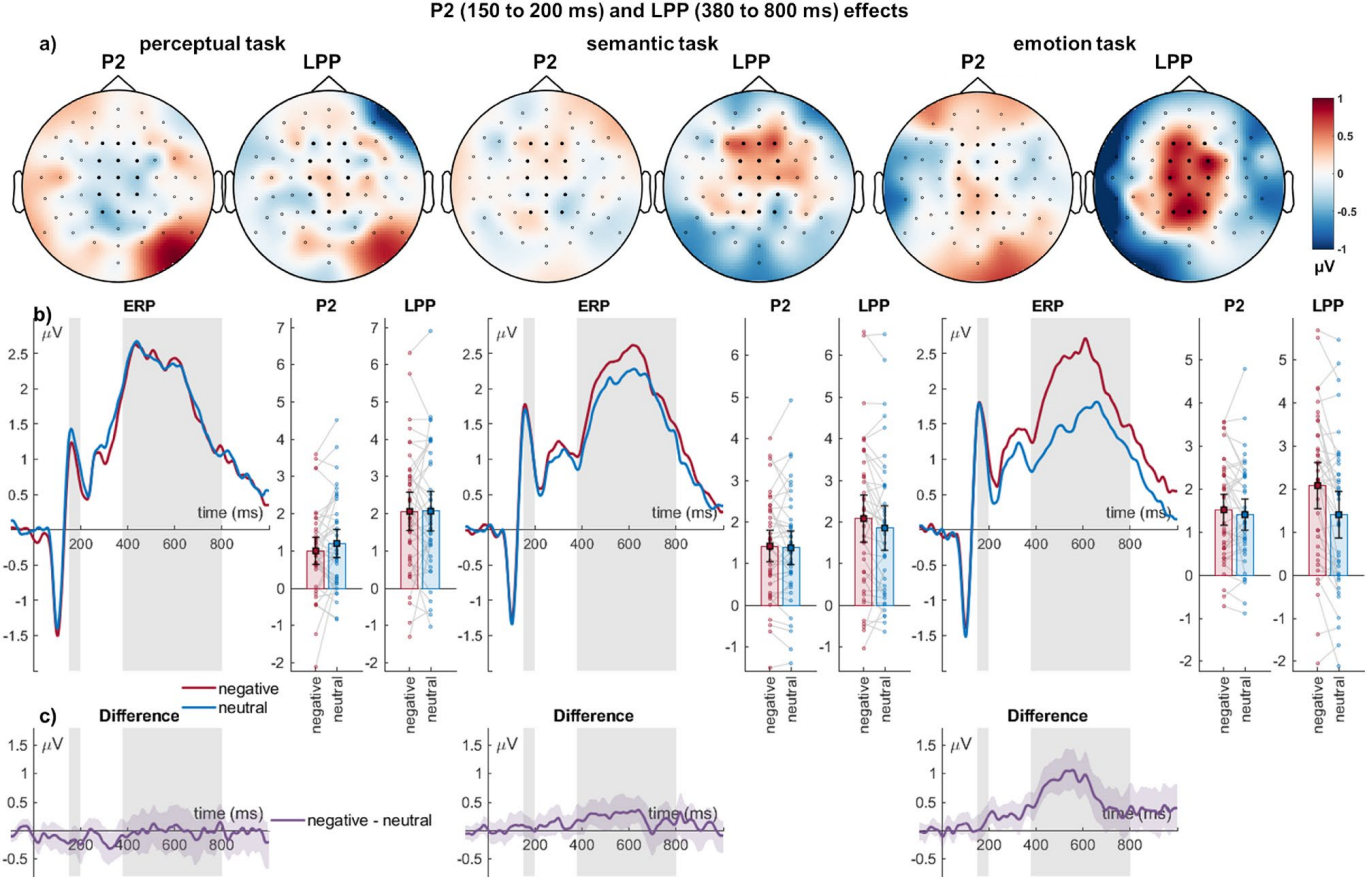
Central cluster showing P2 and LPP effects. (**a**) Scalp topographies depict the differences between negative and neutral words. (**b**) ERP waveforms show the time course over highlighted sensors. For bar plots, error bars show 95% confidence intervals for amplitudes averaged across selected sensors. Lines connect individual data points. (**c**) Respective difference plots displayed below contain 95% bootstrap confidence intervals of intra-individual differences.

For the LPP, there was a main effect of emotion (*F*_(1,39)_ = 11.38, *p* = 0.002, η_P_^2^ = 0.226), with a larger positivity for negative than neutral words. There was no effect of task (*F*_(2,78)_ = 2.09, *p* = 0.131, η_P_^2^ = 0.051). There was an interaction of emotion and task (*F*_(2,78)_ = 6.55, *p* = 0.002, η_P_^2^ = 0.144, see Fig. 3). Importantly, emotion differences were larger for the emotion compared to the perceptual task (*M*_difference_ = 0.68, *SD* = 1.27, *t*_(39)_ = 3.38, *p* = *0.0*02), and for the emotion compared to the grammatical task (*M*_difference_ = 0.45, *SD* = 1.04, *t*_(39)_ = 2.74, *p* = *0.0*09). Negative-neutral differences did not differ between the grammatical and perceptual tasks (*M*_difference_ = 0.23, *SD* = 1.30, *t*_(39)_ = 1.12, *p* = *0.2*70).

### Analyses of hemispheric differences in early emotion effects

#### Hemispheric differences for P1 effects

**Figure 4:**
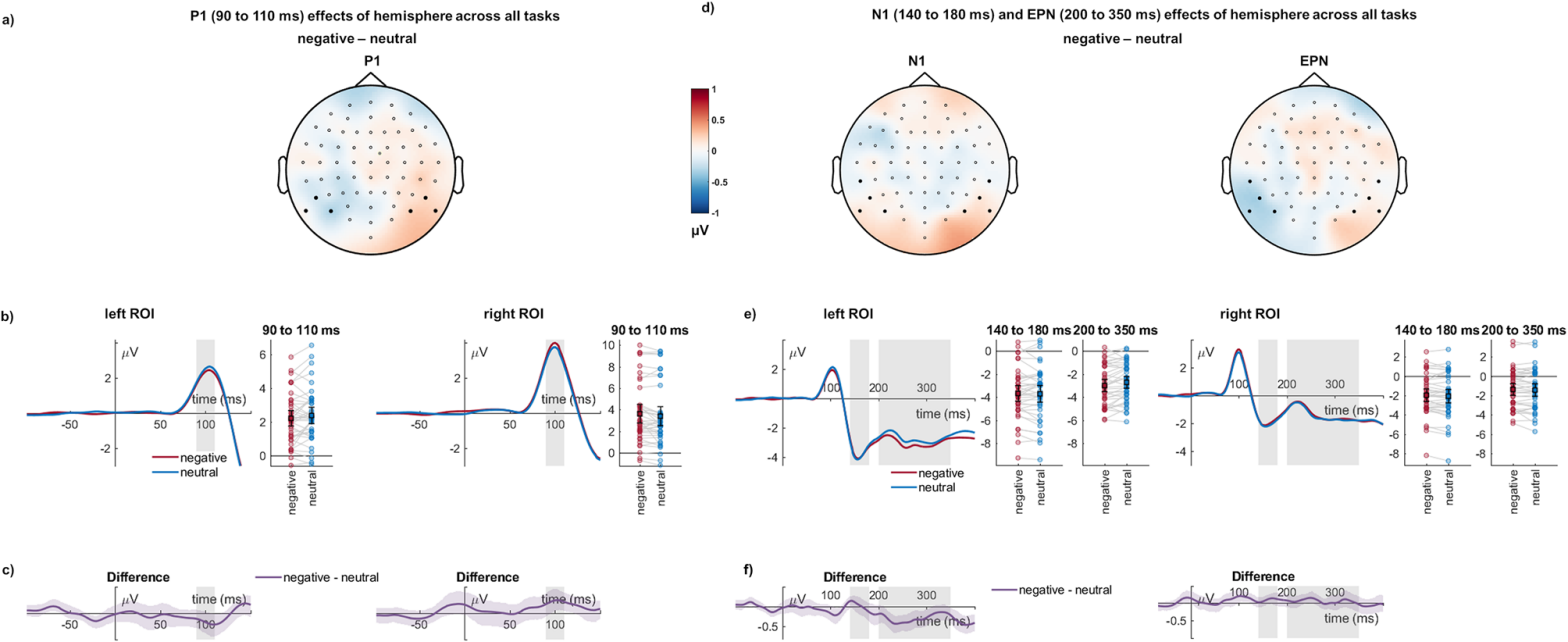
Occipito-temporal clusters, showing P1, N1, and EPN emotion effects across tasks for the left and right hemispheres. (**a**) and (**d**) Scalp topographies depict the differences between negative and neutral words. (**b**) and (**e**) ERP waveforms show the time course over highlighted sensors. For bar plots, error bars show 95% confidence intervals for amplitudes averaged across selected sensors. Lines connect individual data points. (**c**) and (**f**) Respective difference plots displayed below contain 95% bootstrap confidence intervals of intra-individual differences.

For the P1, a main effect hemisphere was found with larger P1 amplitudes over right compared to left sensors (*F*_(1,39)_ = 7.42, *p* = 0.010, η_P_^2^ = 0.160). Hemisphere interacted with emotion effects (*F*_(1,39)_ = 6.85, *p* = 0.013, η_P_^2^ = 0.149, see Fig. 4). Post-hoc tests showed no significant differences over left sensors (M_Difference_ =  − 0.167, *SEM* = 0.107, *p* = 0.127) but significant differences over right sensors (M_Difference_ = 0.223, *SEM* = 0.098, *p* = 0.029), with a larger P1 amplitude for negative words. There was no three-way interaction between hemisphere, emotion, and attention task (*F*_(2,78)_ = 1.71, *p* = 0.186, η_P_^2^ = 0.042).

#### Hemispheric differences for N1 effects

For the N1, a main effect hemisphere was found with larger N1 amplitudes over left compared to right sensors (*F*_(1,39)_ = 13.39, *p* < 0.001, η_P_^2^ = 0.256). Hemisphere did not interact with emotion effects (*F*_(1,39)_ = 0.39, *p* = 0.536, η_P_^2^ = 0.010). There was no three-way interaction between hemisphere, emotion, and attention task (*F*_(2,78)_ = 1.15, *p* = 0.323, η_P_^2^ = 0.029).

#### Hemispheric differences for EPN effects

For the EPN, a main effect hemisphere was found with larger EPN amplitudes over left compared to right sensors (*F*_(1,39)_ = 33.11, *p* < 0.001, η_P_^2^ = 0.459). Hemisphere interacted with emotion effects (*F*_(1,39)_ = 9.31, *p* = 0.004, η_P_^2^ = 0.193, see Fig. 4). Post-hoc tests showed significant differences over left sensors (M_Difference_ =  − 0.279, *SEM* = 0.090, *p* = 0.004), with larger EPN amplitude for negative words, but no significant differences over right sensors (M_Difference_ = 0.065, *SEM* = 0.073, *p* = 0.379). There was no three-way interaction between hemisphere, emotion, and attention task (*F*_(2,78)_ = 2.04, *p* = 0.137, η_P_^2^ = 0.050).

## Discussion

This study investigated how different tasks affect different ERP components to negative vs. neutral words to test whether the dissociation of early, mid-latency, and late emotional differentiation depends on the attended feature of the stimulus. The tasks varied the attentional focus on word-irrelevant features, emotion-irrelevant aspects, or emotional meaning of words. Our findings reveal a systematic pattern of emotional sensitivity varying with the temporal hierarchy of different ERPs, comparable to those observed for other visual, emotional stimuli. We observed interaction effects between emotion and task for the EPN, and LPP, showing an increase of EPN emotion differences during the grammatical and emotion task, while LPP emotion differences were restricted to the emotion task. For the P1, N1, and P2 we observed no main effects of emotion and no interactions between emotion and task, while unregistered control analyses showed reversed P1 emotion effects over right versus left sensors.

These right-lateralized P1 effects should be interpreted cautiously since other studies observed the opposite pattern^23^, and most studies do not observe P1 increases for negative words^12,25,27,28,30,31^, or effects of attention tasks^4,26,29^. Taking these considerations into account, we did not find reliable emotion effects on early ERPs. Besides the P1, this concerns the N1. For the N1, effects in previous studies are mixed, with emotion effects in some studies^3,24,33^, but not in others^12,25,30,35^. The conflicting emotion effects across studies may be due to a combination of specific stimuli, task parameters, variable effects in smaller samples, or individual differences, such as differences in morphosyntactic processing^34^. Our study focused on the effects of negative vs. neutral stimuli. Thus, it remains an open question whether there might be findings for positive stimuli, also applying to the findings concerning the P2 component^1,15,29,55^. While we did not observe early emotion effects with a typical word set used in the field, we do not rule out that specific word by emotion stimulus conditions (e.g., word frequency, word length, concreteness, stimulus presentation duration) might exist, which should be addressed in future high powered studies^12^. Furthermore, early effects might be evident in other analytical EEG/ERP data approaches.

We also found no effects for the P2. In the literature, both increased P2 amplitudes for negative words have been observed^31,37,38^, as well as no differences between negative and neutral words^1,40^. Emotion effects are often shown to be larger in concrete words^39^ and have been reported selectively for positive words in some studies^1^. Thus, similar to the P1 and N1 components, we might have missed specific valence effects in this study.

Concerning the generally increased N1 and P2 amplitudes in the grammatical and emotion task, this could reflect more elaborated processing of the word meaning through top-down instructions^56^. This would align with the observed larger reaction times in the latter two tasks. Please note that we did not register to interpret these general amplitude changes.

In contrast to the earlier components of the ERP, we formulated preregistered hypotheses regarding the EPN and and the LPP. Findings supported the outlined expectations. Our study found no EPN effects when participants attended to the lines. This might be surprising, given that EPN effects are frequently reported across various tasks^2,46,48^. We reason that the absence of emotional differentiation is due to combining a brief presentation duration with the attention directed to the line orientation. Longer durations might also enable participants to decode the emotional information. Brief stimulus durations are necessary to ensure the relevant task focus and avoid additional cognitive processes^51,52,54^. In contrast to the perceptual task, when participants attended to the word meaning or were explicitly asked to evaluate the emotional meaning of the words, larger EPN amplitudes were observed for negative compared to neutral stimuli. Our findings suggest that such differential processing is attenuated or abolished when participants attended to perceptual information, in line with some previous findings^4,44^, but see^45^. Additional analyses with the hemisphere as a factor showed a left-lateralization of EPN emotion modulations in line with the literature^2,17^. The EPN has been suggested to signal early attentional selection^41^, which typically is increased by emotionally (arousing) stimuli^57,58^ but also increased for other salient stimuli^59^. While the EPN was originally thought to be generated in the primary and secondary visual cortex, picture-wise correlation approaches showed stronger correlations with subcortical structures, including the amygdala, anterior cingulate cortex, or the striatum^60,61^. However, studies using separate or combined EEG/fMRI recordings that enable the localization of EPN generators in word stimuli are missing.

In contrast to earlier ERPs, a differential LPP effect for negative words was only seen during the emotion task. In line with this finding, several studies report late effects during tasks that require the processing of the emotion^37,44,46–48^. Further, late emotion effects were larger when participants attended to the emotion but compared to perceptual^37^, or semantic (touchable/not touchable) features^45^. While late effects are generally smaller in verbal stimuli, these are reliable during explicit emotion decision tasks but during non-emotional tasks, such as watching, reading, or classification according to non-emotional attributes^49^. Thus, these findings support the idea that during the LPP stage, evaluative and controlled attention processes occur^20,62^. The LPP likely results from multiple and distributed sources^63^. It has been suggested that these include visual cortices, temporal cortices, the amygdala, the insula, and the orbitofrontal cortex^63^. Stimulus-specific effects can be expected, as for emotional words, fMRI studies show that different frontal regions (inferior frontal gyrus, dorsomedial prefrontal, and cingulate cortex) are involved^39,64^. Studies with suited designs are needed to disentangle the differential involvement of brain areas in the generation of the EPN and the LPP.

Our findings further support the general notion that at least the dynamics and the functional significance of EPN and LPP effects are highly similar across different visual stimulus categories, scenes, faces, or words. For reviews, see^6,42,65–67^. In most studies, EPN and LPP effects are highly correlated^60^, while our attention task manipulation enables a clear dissociation. The findings are remarkably similar to our recent studies with other stimulus categories^51–53^. For example, we found that faces and scenes elicited increased N1/N170 modulations for negative stimuli were task-independent, while an EPN effect was not observed during the perceptual task but found in similar amplitude for the other two tasks^51,52^. LPP differences were only present when attention was directed to the emotional expression of the face^51,52^. Thus, a similar task dependency can be shown across different categories of emotional stimuli, at least for the EPN and the LPP.

## Limitations and future directions

Concerning our study's findings, some constraints have to be mentioned. We only focused on comparing ERPs to negative and neutral words. Future studies might investigate whether findings depend on valence and/or arousal. While positive words would be interesting to examine, several reasons led to the inclusion of only negative and neutral words. First, we used a design comparable to other recent studies focusing on negative versus neutral stimuli^51,52^. Secondly, we aimed to have a similar two-forced choice task in all three tasks. An additional differentiation of positive words would likely increase the difficulty of the emotion task. However, future studies might also use other word stimulus sets or systematically vary stimulus features to better understand possible early effects and include positive words. Furthermore, we would like to note that the dissociation between ERP components requires brief presentation times to avoid attentional spillover to task-irrelevant stimulus features. Here, the brief presentation durations ensured that the participants' attention was only directed at a specific task, showing successful modulations of emotional effects across tasks, similar to previous studies. Nevertheless, future studies might use tasks with varying presentation durations to test whether effects differ with longer presentation times^54^. Finally, we used the collapsed conditions or collapsed differences to identify ERP components of interest. We decided to predefine our ERPs of interest (see methods section) based on studies with similar tasks but different visual stimuli^51,52^. However, other methods can also be used to identify ERPs without biases and may result in different time windows or sensors.

## Conclusion

We found no evidence of early (P1, N1, and P2) ERP differences between negative vs. neutral words across three different attention tasks. However, we observed task-sensitive mid-latency (EPN) and late (LPP) differential processing of emotional words. EPN effects required attention to the word's meaning, while the LPP effect was only seen during the emotional task. These findings reveal a systematic pattern of emotional sensitivity varying with the temporal hierarchy of different components of the ERP, showing the graded increase in processing steps depending on the participants` task set.

## Methods

### Participants

In total, forty-eight participants enrolled in this study and provided complete data. Eight participants were excluded due to EEG rejection criteria, defined as more than 10 interpolated electrodes or more than 40% of usable trials rejected. All participants provided written informed consent to participate in the study. Participants received 10 Euros per hour for participation. The final sample of forty participants (30 female), exhibited a mean age of 23.58 years (*SD* = 3.95, Median 23, *Min* = 18; *Max* = 35), all had normal or corrected-to-normal vision, were right-handed, native German speakers, with no reported history of psychiatric disorders. We followed the updated data-sampling plan and collected 40 usable datasets (see https://osf.io/nrmsb), based on power calculations from a recent previous study using the same attention tasks for faces^51^. Our sample size exhibited a power of > 99% to detect the previously observed large effect sizes (η_p_^2^ = 0.149 and 0.155) for the EPN and LPP interactions. Participants performed an unrelated auditory attention task first, and participation in this task was optional. Data were uploaded to the attached OSF project (https://osf.io/eyndu/). The University of Münster medical ethics committee has approved the study protocol. All experiments were performed in accordance with relevant guidelines and regulations of the University of Münster.

### Stimuli

The words were taken from previously collected rating datasets, rated by students regarding valence and arousal, and matched for linguistic variables. For the experiment, 60 negative and 60 neutral words (30 nouns, 30 adjectives) were used, differing in valence and arousal (see Table 3). Word length and word frequency strongly affect word processing; shorter and more frequent words are processed more quickly^68–71^. Secondly, many (high-frequent) orthographic neighbors have been argued to elicit lateral inhibitory mechanisms at a lexical level^72^. Lines were overlaid to the words using presentation (www.neurobehavioralsystems.org), showing three horizontal or vertical lines (horizontal lines 2 lengths; vertical lines 0.7 lengths; thickness 0.01; centered around x = 0, y = 0, RGB color words 0,0,0; RGB color lines 47,79,79).

**Table 3:** Comparisons of negative and neutral words by One-Way-ANOVAs.

Variable	Negative adjectives	Neutral adjectives	Negative nouns	Neutral nouns	F (3,119)
Valence	2.77^a^	5.09^b^	2.63^a^	5.13^b^	125.42***
	(0.54)	(0.60)	(1.01)	(0.44)	
Arousal	5.74^a^	3.53^b^	5.90^a^	2.45^c^	97.43***
	(1.13)	(0.61)	(1.13)	(0.79)	
Word length	7.90	7.93	7.80	7.80	0.05 n.s
	(1.83)	(1.68)	(1.50)	(1.16)	
Word frequency (per million)	866.97	869.57	876.03	883.30	< 0.01 n.s
	(826.95)	(592.86)	(1211.61)	(945.56)	
Familiarity (absolute)	11,338.27	14,363.63	6913.80	6716.20	0.72 n.s
	(29,043.37)	(35,036.45)	(11,054.46)	(9483.73)	
Regularity (absolute)	91.13	104.63	94.93	126.83	0.22 n.s
	(247.53)	(238.44)	(100.61)	(97.49)	
Neighbors Coltheart (absolute)	3.03	3.70	4.70	3.10	0.77 n.s
	(2.51)	(3.48)	(5.50)	(6.60)	
Neighbors Levenshtein (absolute)	5.50	7.27	8.60	7.50	1.08 n.s
	(4.55)	(5.38)	(7.30)	(8.99)	

All categories contained 30 adjectives/nouns each. *** = *p* ≤ 0.001. Standard deviations appear in parentheses below means; means in the same row sharing the same superscript letter do not differ significantly from one another at *p* ≤ 0.05 based on LSD test post-hoc comparisons.

### Procedure

**Figure 5:**
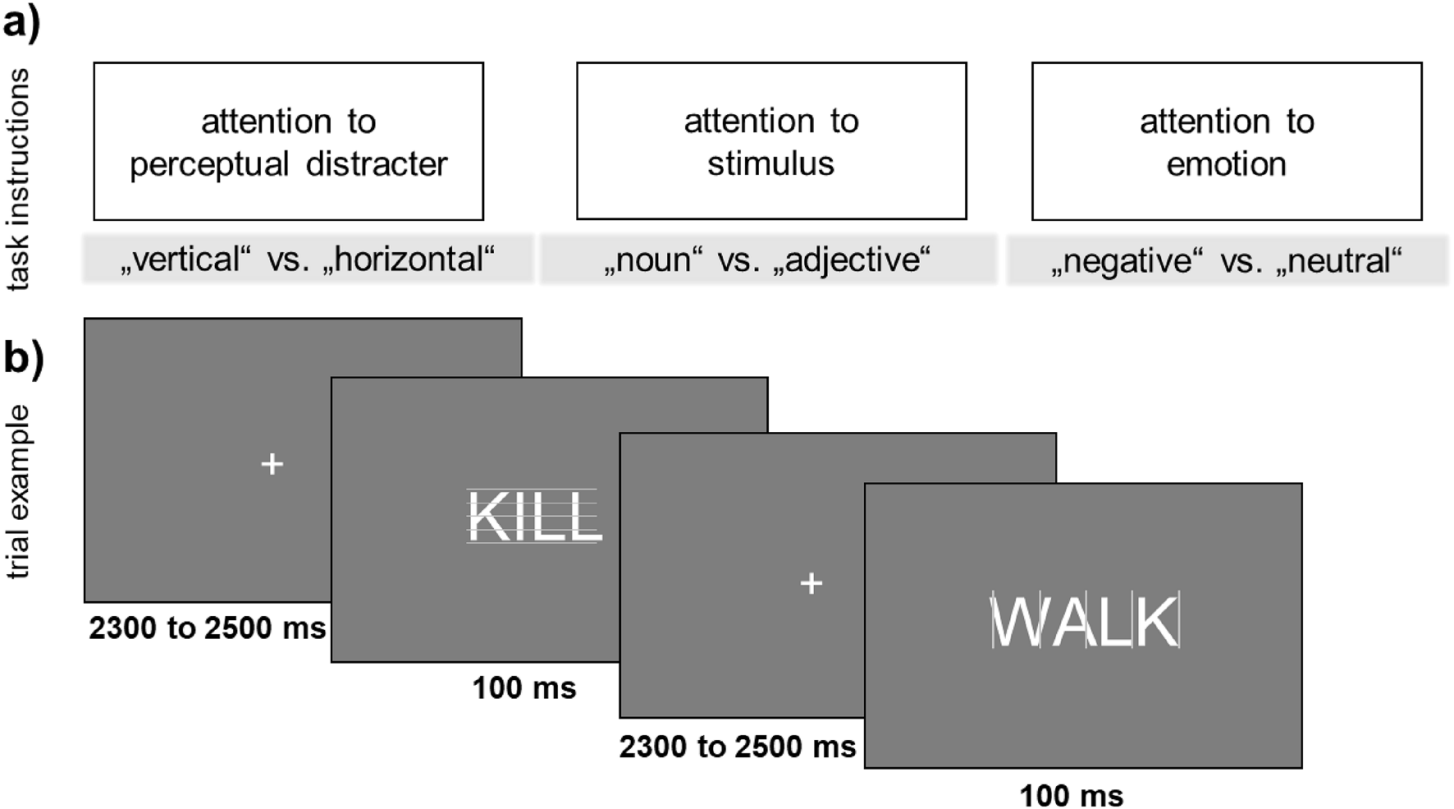
Experiment and trial overview. (**a**) Overview of the three attention tasks. (**b**) Trial structure for each of the three attention tasks. Please note that screen proportions were increased to increase visibility.

Participants were instructed to avoid eye-movements and blinks during the stimulus presentation. While participants were prepared for the EEG, they responded to a demographic questionnaire. They started with either the perceptual decision, grammatical decision, or emotion decision task. Each task contained a block of 120 trials, with all 60 negative and 60 neutral words. The trial structure and presentation were identical (see Fig. 5). In each trial, a word was presented for 100 ms. Afterwards, a variable fixation cross was presented for 2300–2500 ms.

Responses were recorded within the first 1500 ms. Task order and response buttons (x and m) were counterbalanced. In each task, participants always had to decide in a two-alternative forced-choice task: (1) if line orientation was horizontal or vertical, or (2) if the word was a noun or adjective, or (3) if the word was negative or neutral. All 60 negative and 60 neutral words were presented in each task, summing up to a total of 360 trials.

### EEG recording and preprocessing

EEG signals were recorded from 64 BioSemi active electrodes using Biosemis Actiview software (www.biosemi.com). Four additional electrodes measured horizontal and vertical eye-movement. The recording sampling rate was 512 Hz. Offline data were re-referenced to average reference, and band-pass filtered from 0.01 to 40 Hz. Recorded eye-movement was corrected using the automatic eye-artefact correction method implemented in BESA^73^. The remaining artifacts were rejected based on an absolute threshold (< 120 µV), signal gradient (< 75 µV/∂T), and low signal (i.e., the *SD* of the gradient, > 0.01 µV/∂T). Noisy EEG sensors were interpolated using a spline interpolation procedure. A delay of the LCD screen for stimulus presentation of 15 ms, measured by a photodiode, was corrected during epoching. We included only participants with at least 15 correct trials in each condition (see https://osf.io/nrmsb). On average, 47 trials were kept per single condition (*Ms* = 45 to 49, *SDs* = 7 to 8; *Min* = 29 to 32), with no differences between emotion or task conditions and with no interaction (*Fs* < 2.33, *ps* > 0.122). Filtered data were segmented from 100 ms before stimulus onset until 1000 ms after stimulus presentation. Baseline correction used the 100 ms before stimulus onset.

### Data analyses

**Figure 6:**
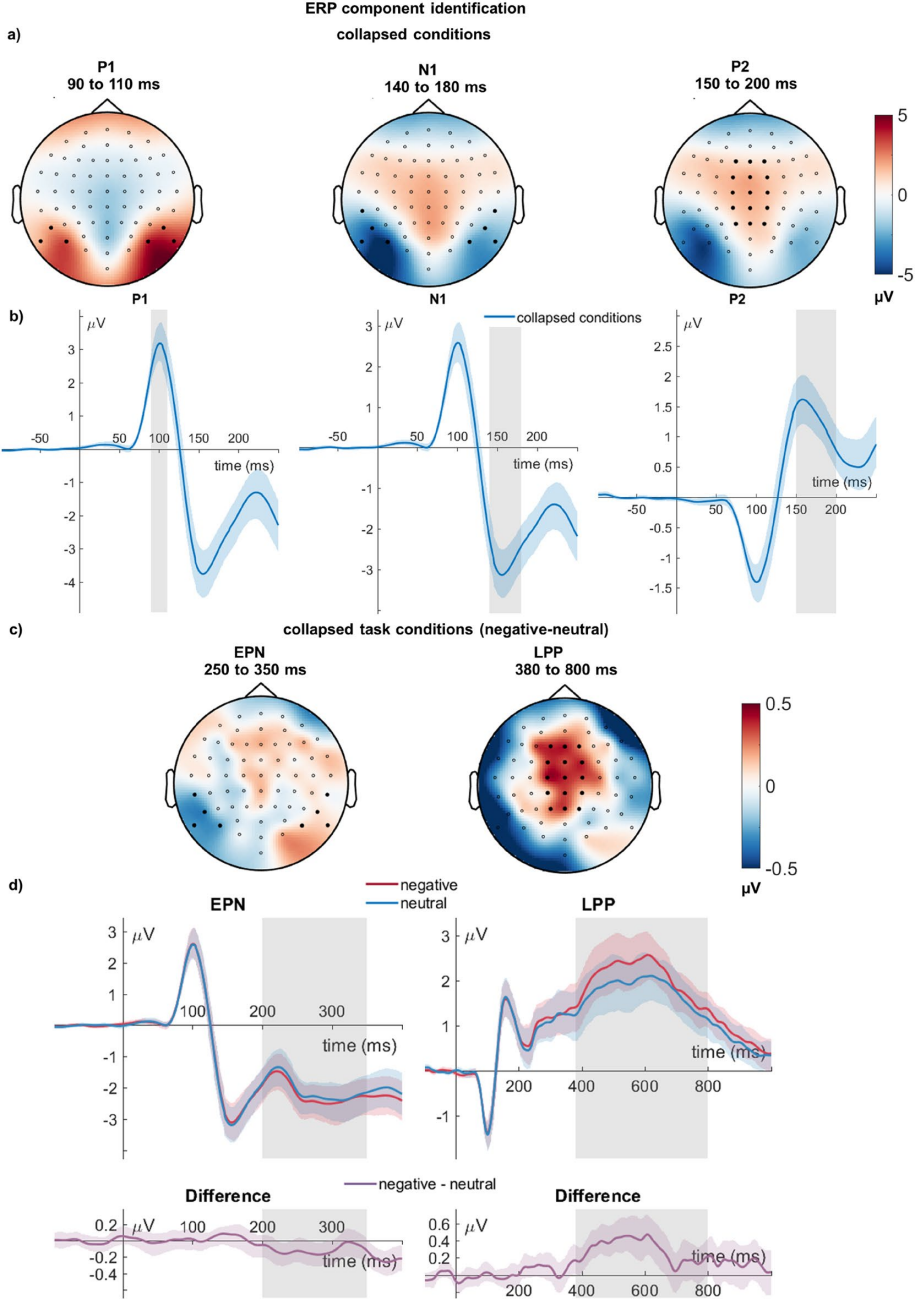
ERP identification validation. (**a**) Collapsed conditions to identify the P1, N1, and P2 components. (**b**) Respective waveforms averaged across highlighted selected sensors. (**c**) Collapsed task conditions to identify the EPN and LPP components. (**d**) Respective waveforms averaged across highlighted selected sensors.

All data were statistically analyzed with two (Emotion: negative, neutral) by three (Task: perceptual, grammatical, emotion decision) repeated measure ANOVAs. We investigated the main effects of task and emotion and their interaction. Partial eta-squared (partial η^2^) were used to describe effect sizes, where η_P_^2^ = 0.02 describes a small, η_P_^2^ = 0.13 a medium and η_P_^2^ = 0.26 a large effect^74^. Behavioral data were analyzed with JASP (https://jasp-stats.org) for both reaction time and accuracy. We predefined expected time windows and sensor clusters, with a validation based on inspection of the waveforms. Using these time windows and scalp regions as priors, the P1, N1, and P2 were identified using a collapsed localizer across all conditions, for which we used the predefined sensors and time windows. Similarly, negative-neutral differences were used for the EPN and LPP (see Fig. 6 for the ERP identification results). Based on the scalp topography, we visually examined whether sensors adequately capture the collapsed positivity/negativity, where the scalp differences for LPP led to a modification of the included sensors (see below). In a second step, we averaged the ERP waveform to visually judge whether the time windows were symmetrically around the positive or negative peak or captured the emotion differences in cases of the EPN and LPP. This led to slight deviations from the registration for the used time windows and sensors. We identified the P1 from 90 to 110 ms (registered 80–100 ms), the N1 from 140 to 180 ms (registered 140–190 ms), the P2 from 150 to 200 ms (registered), the EPN from 200 to 350 ms (registered), and the LPP from 380 to 800 ms (registered 400–650 ms). We could not clearly identify a centro-parietal P3. We averaged ERPs from all examined sensors in the above-defined time windows. We used occipital sensors for the P1, and occipito-temporal sensors for the N1 and the EPN (P1: P9, P7, PO7, P10, P8, PO8; N1 and EPN: TP7, P9, P7, PO7, TP8, P10, P8, PO8). The central cluster (P2, LPP) was examined over an extended sensor cluster (registered C1, Cz, C2, CP1, CPz, CP2, additionally including F1, Fz, F2, FC1, FCz, FC2, P1, Pz, P2). In addition, unregistered analyses tested for hemispheric differences in early emotion effects and possible lateralized interactions between emotion effects and the attention task (P1, N1, EPN).

## Data Availability

All data are available on the Open Science Framework (https://osf.io/eyndu/).
